# Electrocardiographic T-wave Abnormalities and Premature Ventricular Contraction Burden in Patients With Palpitations: A Regional Study From Northeast India

**DOI:** 10.7759/cureus.85629

**Published:** 2025-06-09

**Authors:** Rajeev Bharadwaj, Suman Kalita, Rudrakshya Sahu, Saif Ahmad, Durlav Banskota, Suraj Mungase

**Affiliations:** 1 Department of Cardiology, All India Institute of Medical Sciences, Guwahati, Guwahati, IND; 2 Department of Medicine, All India Institute of Medical Sciences, Guwahati, Guwahati, IND; 3 Department of Surgery, University Hospitals Birmingham NHS, Birmingham, GBR; 4 Department of Maxillofacial Surgery, Royal Stoke University Hospital NHS, Stoke-on-Trent, GBR; 5 Department of Pharmacy Practice, National Institute of Pharmaceutical Education and Research, Guwahati, IND

**Keywords:** ecg, holter, pvc burden, repolarization abnormalities, tp -te interval

## Abstract

Background: Premature ventricular contractions (PVCs), often presenting as palpitations, are common in cardiology outpatient settings. While a high PVC burden is linked to adverse cardiac outcomes, its detection typically requires 24-hour Holter monitoring - an investigation not always feasible in resource-limited settings. Electrocardiographic (ECG) markers of ventricular repolarization, represented by T-wave abnormalities, such as Tp-Te interval, Tp-Te/QT ratio, and QTc dispersion, may serve as accessible surrogate indicators of PVC burden.

Objective: To evaluate the correlation between surface ECG-derived repolarization markers and PVC burden and determine which parameters best predict higher PVC burden in patients presenting with palpitations.

Methodology: A total of 87 adult patients with palpitations referred to a tertiary care cardiology clinic in Northeast India underwent 12-lead ECG and 24-hour Holter monitoring. Patients were stratified into two groups based on PVC burden (<1% vs. >1%). ECG parameters, including T-wave axis angle, frontal QRS-T (fQRS-T) angle, Tp-Te interval, Tp-Te dispersion, Tp-Te/QT ratio, and QTc dispersion, were compared. Statistical analysis included Welch’s t-test and Spearman’s correlation.

Results: Tp-Te interval (*r* = 0.5), Tp-Te/QT ratio (*r* = 0.4), and QTc dispersion (*r* = 0.3) demonstrated significant positive correlations with total PVC burden. These parameters were also significantly higher in the >1% PVC burden group (*P* < 0.05). No significant correlation was observed with T-wave axis or fQRS-T angle.

Conclusions: Among surface ECG markers studied, Tp-Te interval, Tp-Te/QT ratio, and QTc dispersion showed the strongest correlation with PVC burden in patients presenting with palpitations. These accessible and non-invasive markers may help identify patients at risk of higher PVC loads who may benefit from further evaluation, especially in settings where Holter monitoring is limited.

## Introduction

Repolarization abnormalities mainly manifest in the form of premature ventricular contractions (PVCs), which result in palpitations, causing a range of issues, starting from an irritating benign symptom to cardiomyopathy and heart failure in severe cases, also leading to sudden cardiac arrest in rare instances. In available literature, PVC burden of more than 10% suggests development of pathological changes of cardiomyopathy, which may end up in heart failure and are grounds for treatment [1,2,3]. Detection of PVC burden requires at least a 24-hour Holter monitoring, which is usually not found in resource-limited settings, and interpretation of such requires expert knowledge.

Among the markers of Ventricular repolarization on 12-lead surface ECG, Tp-Te ratio, QTc dispersion, and Tp-Te/QT ratio are considered markers of transmural dispersion of repolarization in the left ventricle. Prolongation of these parameters makes one prone to ventricular arrhythmias and sudden cardiac death [4].

In this study, we tried to find whether a simple 12-lead surface ECG can detect the need for further investigation and cardiology follow-up in patients presenting with palpitations, which is a routine cause for cardiology referrals, especially in outpatient consultations.

The study aimed to find out which among the surface ECG parameters of T-wave axis abnormality (T-wave axis angle, frontal QRS -T angle, Tp-Te interval, Tp- Te (d), Tp-Te/QT, and QTc dispersion) best correlates with the PVC burden of the patient and can be used as a surrogate marker to determine PVC burden.

## Materials and methods

A total of 110 consecutive adult patients with complaints of palpitation who were referred for cardiology consultation were seen by a cardiologist in a tertiary care center in Northeast India. Among them, 87 were advised for 24-hour Holter monitoring based on suspicion of an arrhythmic cause of the palpitation. Patients with previous myocardial infarction (MI), bundle branch blocks, permanent atrial fibrillation, hypertrophic cardiomyopathy (HCM), electrolyte imbalance, thyroid disorder, permanent pacemaker, and on chronic drug therapy (pro- or antiarrhythmics) were excluded. Informed consent was taken from all the participants for the study. Their baseline 12-lead ECG was recorded at the time of referral in resting state, and a 24-hour Holter recording was interpreted using Hscribe Version 6.4.0 software (Welch Allyn, Inc., Skaneateles Falls, NY) and reported by a cardiologist.

Baseline ECG parameters were obtained as described below.

The patients were divided into two groups based on their 24-hour PVC burden: less than 1% (<1% group) and greater than 1% (>1% group). The following ECG parameters were compared:

The T-wave axis angle was recorded from the ECG. A normal T-wave axis angle ranges from 15° to 75° [5].

The frontal QRS-T (fQRS-T) angle was calculated from a standard 12-lead ECG as the absolute difference between the frontal plane QRS axis and T-wave axis. If such a difference exceeded 180°, then the frontal QRS-T angle was calculated as 360° minus the absolute value of the difference between the frontal plane QRS axis and T-axis [6].

The Tp-Te interval was measured from the peak of the T-wave to the end of the T-wave. The end of the T-wave was defined as the intersection of the tangent to the down slope of the T-wave and the isoelectric line [7]. The QT interval was measured from the earliest onset of the QRS complex to the end of the T-wave. When a U wave interrupted the T wave before returning to baseline, the interval was measured to the nadir of the curve between the T and U waves. The Tp-Te/QT ratio was calculated as the ratio of Tp-Te in lead V5 to the corresponding QT interval in the same lead [8]. The Tp-Te and QT intervals were measured in lead V5 [8,9]. If V5 was not suitable, leads V4 and V6 in that order were measured [10]. If the T-wave amplitude was <1.5 mm in a particular lead or morphologically distorted, that lead was excluded from analysis.

T-peak-to-T-end dispersion was defined as the difference between the maximum and minimum T-peak-to-T-end interval in all ECG leads [11]. QTc dispersion was defined as the difference between the longest and shortest QT intervals and rate corrected using the Fridericia formula, as the resting heart rate was less than 100 in all ECGs [12].

Data were recorded by technicians. The QRS angle, T-wave angle, and QTc interval were measured using software, while the frontal QRS-T angle and the remaining parameters were calculated manually. Observer training was given by the cardiologist.

The Institute's ethics committee approved the study vide reference no. M2/P10/2023 dated September 15, 2023.

Data were analyzed using GraphPad Prism 8.0.2 software, Inc., La Jolla, CA. Descriptive data were tabulated as mean with standard deviation, and groups were compared using an unpaired t-test with Welch’s correction as sample sizes were not equal in both groups. Welch's t-test was used over the traditional Student's t-test because it does not assume equal variances between the groups and adjusts for differences in sample sizes. *P* ≤ 0.05 was considered statistically significant. A heat map of correlation data was also made to showcase the different strengths of correlation using Spearman’s rank correlation coefficient.

## Results

A total of 87 patients were included in the study, and a correlation analysis was conducted between ECG parameters and PVC burden. Of the total participants, 49 patients were in the <1% PVC group and 13 in the >1% PVC group. Twenty-five patients were excluded from the group analysis due to incomplete 24-hour Holter recordings. Baseline anthropometric data are presented as mean ± SD and ratios in Table 1.

**Table 1:** Baseline parameters of patients. SD, standard deviation; b/m, beats per minute

Sr.no	Parameters	Mean ± SD/Ratio
1	Age (years)	54.01 ± 14.23
2	Average heart rate in baseline ECG (b/m)	62.23 ± 13.59
3	Gender (M:F)	71:16

**Figure 1:**
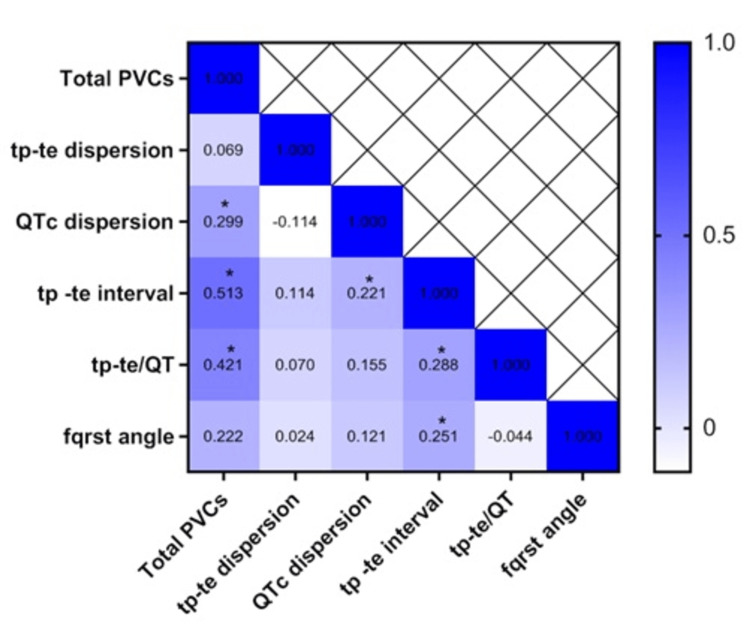
Heat map showing the correlation of parameters. *P < 0.05 was considered statistically significant. Values represent Spearman's correlation coefficient.

A significant overall positive correlation was found between the Tp-Te interval, Tp-Te/QT ratio, and QTc dispersion with total PVC burden, with correlation coefficients of 0.5, 0.4, and 0.3, respectively, as shown in Table 2 and the heat map (Figure 1).

**Table 2:** Overall parameter correlations. *P < 0.05 was considered statistically significant. Values represent Spearman's correlation coefficient. fQRS-T, frontal QRS-T

Parameters	Total PVCs	Tp-Te dispersion	QTc dispersion	Tp-Te interval	Tp-Te/QT	fQRS-T angle
Total PVCs	1.000	0.069	0.299	0.513	0.421	0.222
Tp-Te dispersion	0.069	1.000	-0.114	0.114	0.070	0.024
QTc dispersion	* 0.299	-0.114	1.000	0.221	0.155	0.121
Tp-Te interval	* 0.513	0.114	* 0.221	1.000	0.288	0.251
Tp-Te/QT	* 0.421	0.070	0.155	* 0.288	1.000	-0.044
fQRS-T angle	0.222	0.024	0.121	* 0.251	-0.044	1.000

The values of QTc dispersion, Tp-Te interval, and Tp-Te/QT ratio were also significantly higher in the group with a PVC burden greater than 1% compared to the group with a PVC burden less than 1%, as shown in Table 3.

**Table 3:** Comparison of ECG parameters between total PVC <1% and total PVC >1% groups. Data are expressed as mean ± SD, and groups were compared using an unpaired t-test with Welch’s correction. *P ≤ 0.05 was considered statistically significant. ms, millisecond; SD, standard deviation; PVC, premature ventricular contraction; ECG, electrocardiographic; fQRS-T, frontal QRS -T

Parameter	Total PVC <1% (*n* = 49)	Total PVC >1% (*n* = 13)	*P*-value	*t*-value
Tp-Te dispersion (ms)	1.85 ± 0.70	2.15 ± 0.89	0.2861	1.103
QTc dispersion (ms)	80.08 ± 26.99	108.8 ± 32.75	0.0098	2.915
Tp-Te interval (ms)	78.43 ± 12.50	98.31 ± 16.55	0.0010	4.036
Tp-Te/QT	0.19 ± 0.024	0.27 ± 0.044	<0.0001	5.954
fQRS-T angle (degrees)	57.67 ± 53.71	86.38 ± 59.61	0.1331	1.575
QTc interval (ms)	401.4 ± 31.96	429.0 ± 38.61	0.0303	2.368

**Figure 2:**
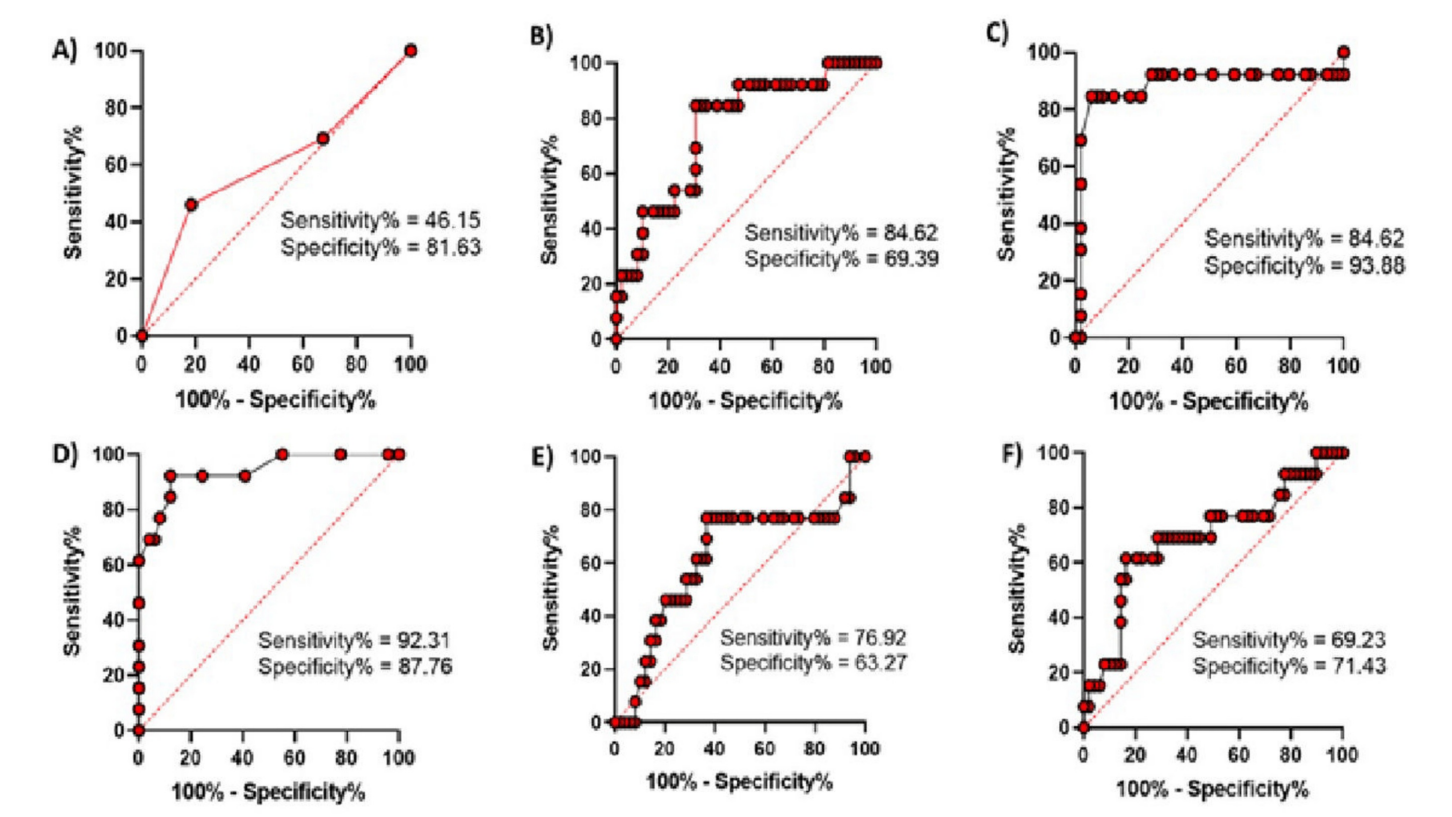
ROC curve analysis of ECG parameters: (A) Tp-Te dispersion, (B) QTc dispersion, (C) Tp-Te interval, (D) Tp-Te/QT ratio, (E) fQRS-T angle, and (F) QTc interval. ROC, receiver operating characteristic; AUC, area under the curve; fQRS-T, frontal QRS-T; ECG, electrocardiographic

Among the measured ECG parameters, the Tp-Te/QT ratio demonstrated the best diagnostic performance, with an area under the curve (AUC) of 0.9388 (95% confidence interval (CI): 0.86-1.00), sensitivity of 92.31%, specificity of 87.76%, and a highly significant *P*-value (<0.0001). This reflects outstanding discrimination between groups, as AUC values >0.90 are regarded as excellent for clinical practice. The Tp-Te interval was also very well-performing diagnostically, with an AUC of 0.8823 (95% CI: 0.73-1.00), sensitivity of 84.62%, specificity of 93.88%, and a *P*-value <0.0001, indicating strong clinical usefulness. QTc dispersion also possessed significant diagnostic value, with an AUC of 0.7669 (95% CI: 0.62-0.90), sensitivity of 84.62%, specificity of 69.39%, and a statistically significant *P*-value (0.0033). This puts it in the "fair" range for clinical usefulness. QTc interval had an AUC of 0.6915 (95% CI: 0.51-0.86), with sensitivity of 69.23%, specificity of 71.43%, and a *P*-value of 0.0349, which suggests poor to fair diagnostic performance. The Tp-Te dispersion and fQRS-T angle both had lower AUC values (0.6013 and 0.6217, respectively), with the associated sensitivities and specificities indicating their limited clinical usefulness. The *P*-values for these measures (0.2647 and 0.1802, respectively) were not statistically significant, indicating that their ability to discriminate between groups is unreliable, as shown in Table 4 and Figure 2.

**Table 4:** ROC analysis of ECG parameters. ROC, receiver operating characteristic; AUC, area under the curve; CI, confidence interval; fQRS-T, frontal QRS-T

ECG parameters	AUC value	95% CI	Sensitivity (%)	Specificity (%)	*P*-value
Tp-Te dispersion	0.6013	0.40- 0.79	46.15	81.63	0.2647
QTc dispersion	0.7669	0.62- 0.90	84.62	69.39	0.0033
Tp-Te interval	0.8823	0.73-1.00	84.62	93.88	<0.0001
Tp-Te/QT	0.9388	0.86-1.00	92.31	87.76	<0.0001
fQRS-T angle	0.6217	0.43-0.80	76.92	63.27	0.1802
QTc interval	0.6915	0.51-0.86	69.23	71.43	0.0349

## Discussion

Our study demonstrated that the Tp-Te interval, Tp-Te/QT ratio, and QTc dispersion were significantly positively correlated to the total PVC burden in a 24-hour Holter recording in outpatient consultation of patients complaining of palpitations with or without evidence of PVC in a baseline 12-lead standard surface ECG.

As all these ratios are established markers of myocardial repolarization and can be assessed using a standard 12-lead surface ECG, their relevance was theoretically well-founded. However, to the best of our knowledge, this is the first study to correlate these objective repolarization parameters with clinical symptoms of palpitations in an outpatient cardiology clinic.

Although the correlation coefficients were not very strong, these were statistically significant and positively correlated with a significant difference between the two PVC groups. If the sample size were large, we postulate that the correlation would be much stronger. Nonetheless, the Tp-Te/QT ratio and the Tp-Te interval were the most diagnostically useful ECG parameters in these data, with good AUC values, sensitivities, specificities, and statistically significant *P*-values, showing strong clinical utility potential. QTc dispersion possessed fair utility, while the other parameters possessed limited utility for diagnostic discrimination.

Similar findings in relation to Tp-Te interval and QTc dispersion were found in a study in 2022 in Turkey, which aimed to correlate PVC burden with fQRS-T angle [13].

Our study did not find a statistically significant correlation between fQRS-T and PVC burden, nor between the two groups compared. This may be due to the small sample size and the strong exclusion criteria. Also, a wider fQRS-T angle is more strongly correlated with increased risk of CVDs and mortality [14], which was not specifically our study population or aim, ours being a more generalized heterogeneous study population complaining of only palpitation. None of the study participants exhibited surface ECG findings consistent with long or short QT syndromes, pre-excitation, or Brugada syndrome.

A PVC burden >24 % has been suggested as having the highest sensitivity and specificity (79% and 78%, respectively) to predict the occurrence of PVC-induced cardiomyopathy, but a recent study has also shown that even among those with 3% PVCs, more than half would go on to develop heart failure [15].

Karaman et al. also observed an increase in the Tp-Te interval and Tp-Te/QTc ratio with increasing PVC burden; however, the increase in the Tp-Te/QT ratio was not statistically significant [16]. Similarly, Akdi et al. reported significantly higher Tp-Te intervals, Tp-Te/QT ratios, and fQRS-T angles in patients with a higher PVC burden [17].

In a large cohort study, individuals with a higher frequency of PVCs had similar rates of heart failure as those with isolated PVCs, and both groups had higher rates than those without PVCs. The presence of PVCs was also associated with incident heart failure, independent of underlying coronary heart disease [18].

In 2025, Kramarenko et al. concluded in their study that in the Amsterdam University Medical Center (AUMC) cohort, PVC burden exceeding 0.5%, equal to about 3-4 beats per minute, is significantly associated with increased cardiac event risk [19]. This also justifies the division of our data into PVC burden of more than 1% and less than 1% groups for analysis and has provided valuable insights into surface ECG markers, which can point toward the possibility of increased PVC burden in outpatients complaining of palpitations.

As further research in the morphology of PVC involved in PVC-induced cardiomyopathy is still in the nascent stages, surface ECG parameters in suspected patients vulnerable to higher PVC load are imperative for early diagnosis and prevention of heart failure and associated complications.

Further studies are needed to firmly establish the relationship between surface ECG repolarization markers and 24-hour Holter-derived PVC burden in patients presenting with palpitations suspected to be of arrhythmic origin.

If future studies can establish a significant relationship between these markers of repolarization and clinical assessment, including a 12-lead surface ECG, it could provide a simple, cost-effective assessment technique for the common clinical problem of palpitations, aid in proper management, and help prevent a curable form of heart failure.

Limitations

An important limitation of the study is that, since subject recruitment was based on clinical history, the majority of patients had a total PVC burden of less than 1% over 24 hours. A larger sample size might have yielded a more robust correlation. Additionally, since this was an observational study conducted in a hospital setting with subjects admitted for evaluation, many participants likely limited their usual daily activities, which may have affected the accuracy of the recorded PVC burden. Third, complete 24-hour Holter recordings could not be obtained for 25 patients due to subject-related and technical issues, further reducing the sample size available for comparison between the two groups.

## Conclusions

Among all surface ECG-derived repolarization markers evaluated, Tp-Te interval, Tp-Te/QT ratio, and QTc dispersion were the most strongly correlated with PVC burden in symptomatic patients presenting with palpitations. These easily obtainable parameters may help identify patients who require further evaluation with Holter monitoring, particularly in resource-constrained settings.
